# Relationship between Serum Osteocalcin Level and Gestational Diabetes Mellitus: A Case-Control Study

**DOI:** 10.4314/ejhs.v30i5.6

**Published:** 2020-09

**Authors:** Saloumeh Peivandi, Kamelia Yaghoubinia, Zahra Kashi, Siavash Moradi, Ali Habibi

**Affiliations:** 1 Department of Gynecology and Obstetrics, Faculty of Medicine, Mazandaran University of Medical Sciences, Sari, Iran; 2 Department of Obstetrics and Gynecology, Faculty of Medicine, Ramsar International Branch, Mazandaran University of Medical Sciences, Sari, Iran; 3 Department of Internal Medicine, Faculty of Medicine, Diabetes Research Center, Mazandaran University of Medical Sciences, Sari, Iran; 4 Educational Development Center, Psychiatry and Behavioral Sciences Research Center, Addiction Research Institutes, Mazandaran University of Medical Sciences, Sari, Iran; 5 Student Research Committee, Faculty of Medicine, Mazandaran University of Medical Sciences, Sari, Iran

**Keywords:** Osteocalcin, Diabetes, Gestational, Vitamin D

## Abstract

**Background:**

Osteocalcin (OC) is the most common noncollagenous protein in bone matrix, which is synthesized only in bone tissue and by osteoblasts. The potential role of osteocalcin on glucose and fat metabolism has been previously reported. The aim of this study was to compare the serum OC level in pregnant women with and without gestational diabetes mellitus (GDM).

**Methods:**

In the present case-control study, all pregnant women who were referred to a obstetrics and gynecology clinic in Sari, Iran, and met the inclusion criteria underwent an overall screening with a 75-g glucose tolerance test (GTT) at week 24 to 28 of gestation. The study was conducted between September 2018 and February 2019. Based on criteria, the pregnant women with confirmed GDM were matched with pregnant women without GDM in terms of baseline characteristics such as chronological age and BMI. The serum OC levels were also measured if vitamin D and calcium levels were normal. All data were analyzed using SPSS 21.

**Results:**

The two groups with and without GDM had no significant difference in terms of age, BMI and OC level. There was no significant correlation between age and BMI with OC level in healthy pregnant women, respectively (P=0.49 and P=0.58). The correlation between BMI and age with OC level in GTT-positive pregnant women was 0.05 and -0.172, respectively, which was not significant (P=0.77 and P=0.36).

**Conclusion:**

According to the results of this study, there is no significant difference of serum OC levels in pregnant women with GDM compared to healthy pregnancy. Given that the levels of serum insulin or insulin resistance have not been assessed, these indices are recommended to be evaluated in future studies.

## Introduction

Osteocalcin (OC) is the most common noncollagenous protein in bone matrix, which is synthesized only in bone tissue and by osteoblasts (1). The OC levels reflect directly the bone turnover, whose measurement is strongly correlated with the actual state of bone metabolism. Increased levels of OC are observed in bone diseases associated with increased bone turnover, such as Paget's disease and metastatic cancers (1–3). The OC levels in type II diabetes mellitus (DM) are negatively correlated with increased insulin resistance. The OC levels in patients with type II DM are lower than the healthy individuals (4).

Pregnancy causes many hormonal changes, one of which is increased insulin resistance. Such physiological change aims to ensure continuous supply of fetal glucose in the post-meal phase (5). Gestational diabetes mellitus (GDM) is a disorder induced by pregnancy, and its possible etiology is the exacerbation of physiological changes in glucose metabolism (6). It has been previously shown that the OC levels were significantly higher in the GDM women than in the healthy pregnant women (1,7). However, some studies found conflicting results in serum OC levels were not significantly different between the women with and without GDM during pregnancy and three months postpartum (8–9).

There have been limited studies on the correlation between GDM with glucose tolerance impairment and serum OC levels in pregnant women. Also, the limited available studies so far show conflicting results. Therefore, the exact association between OC levels and glucose metabolism and insulin resistance during pregnancy is still unclear. Accordingly, the aim of this study was to compare the serum OC level in pregnant women with and without GDM.

## Methods

The present case-control study was conducted on a population of pregnant women aged 20–35 years with a body mass index (BMI) of less than 27 referred to the Mostafavian Clinic of Mazandaran University of Medical Sciences from September 2018 to February 2019. According to the study by Winhofer et al. (1), the total sample size was estimated to be 150 (75 in each group). With consecutive sampling, all women who presented to the Obstetrics and Gynecology Clinic were evaluated. The inclusion criteria were pregnant women aged 20–35 years with BMI less than 27 and normal vitamin D and calcium levels. Also, confirmed diagnosis of GDM for case group and not previously identified as having GDM for control group were other inclusion criteria. The exclusion criteria were women with previously confirmed type I or type II DM, women with a history of hypercholesterolemia, hyperuricemia, thyroid disease, hyperparathyroidism, chronic diseases (liver and renal failure), and metastatic cancers. All pregnant women who met the inclusion criteria underwent an overall screening with a 75-g glucose tolerance test (GTT) at week 24 to 28 of gestation. Based on the criteria proposed by the World Health Organization (WHO), the patient was diagnosed with GDM if her fasting blood glucose (FBS) level was ≥92 mg/dl, one-hour GTT was ≥180 mg/dl, or twohour GTT was ≥153 mg. Ten milliliter of fasting venous blood was taken from all subjects. Then, the venous blood sampling was repeated one and two hours after eating 75-g glucose solution.

All pregnant women diagnosed with GDM who were included in the study were matched with pregnant women without GDM in terms of baseline characteristics such as chronological age and BMI. Pre-pregnancy maternal weight was recorded at the first pregnancy visit. All measurements of height, weight and BMI were performed by an obstetrics and gynecology resident. The maternal height was measured using a tape measure in standing position without shoes while the shoulders were in normal condition. The serum OC levels were also measured if vitamin D and calcium levels were normal. The serum Ca levels were measured using complexometric titration, o-Cresolphthalein (Pars test), and the serum vitamin D using enzymatic immunoassay (EIA, DRG Company of America) at the same medical laboratory. In line with ethics and confidentiality, all study participants were informed about the objectives of the study, the confidentiality of the data and the anonymity of the data. In addition, they could freely leave the partnership.

Quantitative data were described with mean (standard deviation) or median (interquartile range) and qualitative data with frequency (percentage). Independent t-test or Mann-Whitney tests were used to analyze the difference in mean serum OC levels between the two groups. P-value<0.05 was considered statistically significant. All data were analyzed using IBM SPSS 21 software.

## Results

There is no significant difference between two groups in terms of demographic characteristics of participants (Table 1).

**Table 1:** Mean and standard deviation of age and BMI in study groups

Variables	GTT status	Mean (SD)	P-value
Age (years)	Non-Diabetic	28.57 (4.42)	0.34
	Gestational Diabetes Mellitus	29.73 (5.02)	
BMI (kg/m^2^)	Non-Diabetic	25.54 (2.73)	0.79
	Gestational Diabetes Mellitus	25.72 (2.49)	
	Gestational Diabetes Mellitus	16.91 (9.68)	

GTT: glucose tolerance test; BMI: Body mass index

The mean (SD) of serum OC level in women with and without GDM was 16.91 (9.68) and 13.58 (5.60), respectively (p=0.10). In this study, we examined the correlation between values of maternal biometric indices and OC levels in women with and without GDM. The correlation between age and BMI with OC level was -0.13 and 0.10 in pregnant women without GDM, respectively, which was not statistically significant (P=0.49 and P=0.58, respectively). Also, the correlation between BMI and age with OC level was 0.05 and -0.17, respectively, which was not significant (P=0.77 and P=0.36, respectively) (Table 2).

**Table 2:** Determination of correlation between osteocalcin level and values of maternal biometric indices in women with and without GDM

Variables	Indices	Status of diabetes	
		
		Negative GTT	Positive GTT
**Maternal age**	Frequency	75	75
	Correlation	-0.13	0.05
	Significance	0.49	0.77
**Maternal body mass** **index**	Frequency	75	75
	Correlation	0.10	-0.17
	Significance	0.58	0.36

GTT: glucose tolerance test

The results of the study showed a non-significant difference of OC level between diet-controlled and diets plus insulin controlled GDM patients (Table 3).

**Table 3:** Serum osteocalcin level in diet-controlled and diet plus insulin controlled women with GDM

Variable	Diet-controlled	Diet plus Insulin controlled	P-Value
**Frequency (percentage)**	19 (25.32%)	56 (74.75%)	0.12
**Serum osteocalcin level (ng/dl)**	17.34±8.56	15.98±8.63	

**Figure 1:**
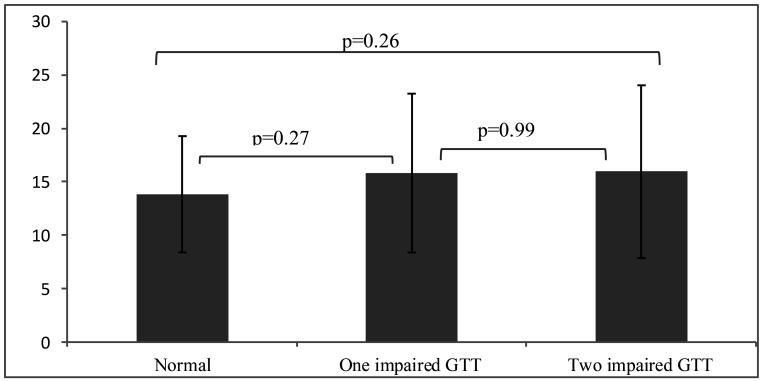
Comparison of serum OC level in pregnant women who had one or two impaired GTT results and healthy pregnant women

According to Figure 1, there is no significant difference between pregnant women who had one impaired GTT result and who had two impaired GTT results in terms of serum OC level (Figure 1).

## Discussion

The results of the present study indicate that the mean serum OC level in women with GDM was higher than the one in the healthy pregnant women. However, the difference was not statistically significant. Also, the serum OC level in pregnant women with one or two impaired GTT result was not significantly different. Additionally, no statistically significant differences were observed between OC levels in diet-controlled GDM patients and patients with diet plus insulin controlled group. In line with the results of our study, Ogueh et al. showed that the GDM had little effect on the serum levels of maternal bone metabolism markers. Even patients with two impaired glucose tolerance test results had no significant difference in their serum OC levels with the group without GDM (10). Srichomkwun et al. found that the serum OC level did not differ between women with one impaired GTT result and women with two or more impaired GTT results, though it was significantly higher than in women with all normal measurements. They found that the serum OC level was significantly and directly correlated with insulin secretion and insulin resistance, but the serum OC level was not significantly different between women with GDM and healthy pregnant women. Also, the mean serum OC level in the healthy pregnant women was not significantly different from the women with GDM. Also, no significant differences of serum OC level were seen in pregnant women with one impaired GTT result and in the pregnant women with two abnormal GTT results (3), which is in line with the results of our study. In the present study, the insulin levels and insulin resistance index were not measured, but similarity was observed in the results of the study.

Saucedo et al. found that the bone biomarkers such as serum OC level were similar in the two groups with and without GDM and were not statistically significant. The serum OC level did not correlate with insulin levels and insulin resistance index. In addition, the serum OC level in the women with GDM who needed insulin did not differ significantly from those on a mere diet. The women with stable postpartum diabetes had lower serum OC levels than the healthy women (8). In a study by Telejko et al. the serum OC level was not significantly different in the patients with and without GDM during pregnancy and three months postpartum. However, in the group examined for OC by polymerase chain reaction (PCR), the patients with GDM had lower OC mRNA expression than the healthy women, probably because adipose tissue is a reduced source of active OC (9). Tabatabaei et al. showed that OC levels were significantly higher in GDM women than in the healthy pregnant women. However, the serum vitamin D levels were not significantly different between the two groups (11). In our study, vitamin D was considered as a confounding factor, and women with normal vitamin D levels were enrolled in the study, which may be one of the strengths of our study. Winhofer et al. found that the serum OC levels were significantly higher in the GDM women than in the healthy pregnant women, although there was no statistical difference between the two groups at 12 weeks postpartum (1). The results of this study contradict with the results of the present study. Smaller sample size and not evaluating vitamin D levels in this study, as a confounding factor, may be a possible explanation for this contradictory results.

One of the strengths of our study was that the levels of vitamin D and calcium were measured in the pregnant women, and the subjects with normal results underwent the measurement of the serum OC levels. In addition, women with a history of hyperuricemia, hyperparathyroidism, chronic liver and kidney disease, and women taking bone metabolism drugs such as corticosteroids and anticonvulsants, as well as those with recent bone fractures were excluded from the study to accurately calculate the serum OC level independent of influencing factors. However, our study has some limitations. We did not investigate the correlation between OC and insulin resistance indices (QUICK1, HOMA2IR, and HOMA1IR) and pancreatic β-cell activity (HOMAB). It might be better to conduct more extensive studies with the above indicators in mind. In conclusion, the results of this study highlighted no significant differences of serum OC levels between pregnant women with and without GDM. Given that the levels of serum insulin or insulin resistance have not been assessed, these indices are recommended to be evaluated in future studies.

## References

[1] Winhofer Y, Handisurya A, Tura A, Bittighofer C, Klein K, Schneider B (2010). Osteocalcin Is Related to Enhanced Insulin Secretion in Gestational Diabetes Mellitus. Diabetes Care.

[2] Starup-Linde J, Vestergaard P (2016). Biochemical bone turnover markers in diabetes mellitus—a systematic review. Bone.

[3] Srichomkwun P, Houngngam N, Pasatrat S, Tharavanij T, Wattanachanya L, Khovidhunkit W (2016). Undercarboxylated osteocalcin is associated with insulin resistance, but not adiponectin, during pregnancy. Endocrine.

[4] Oh JH, Lee NK (2017). Up-regulation of RANK expression via ERK1/2 by insulin contributes to the enhancement of osteoclast differentiation. Mol Cells.

[5] Kovacs CS (2016). Maternal mineral and bone metabolism during pregnancy, lactation, and post-weaning recovery. Physiol Rev.

[6] Eschler DC, Kulina G, Garcia-Ocana A, Li J, Kraus T, Levy CJ (2018). Circulating levels of bone and inflammatory markers in gestational diabetes mellitus. Biores Open Access.

[7] Martinez-Portilla RJ, Villafan-Bernal JR, Lip-Sosa DL, Meler E, Clotet J, Serna-Vela FJ (2018). Osteocalcin serum levels in gestational diabetes mellitus and their intrinsic and extrinsic determinants: systematic review and meta-analysis. J Diabetes Res.

[8] Saucedo R, Rico G, Vega G, Basurto L, Cordova L, Galvan R (2015). Osteocalcin, under-carboxylated osteocalcin and osteopontin are not associated with gestational diabetes mellitus but are inversely associated with leptin in non-diabetic women. J Endocrinol Invest.

[9] Telejko B, Kalejta K, Kuzmicki M, Wawrusiewicz-Kurylonek N, Lipinska D, Pliszka J (2015). The association of bone turnover markers with pro-and anti-inflammatory adipokines in patients with gestational diabetes. Ann Agric Environ Med.

[10] Ogueh O, Khastgir G, Studd J, Jones J, Alaghband-Zadeh J, Johnson MR (1998). Maternal and fetal plasma levels of markers of bone metabolism in gestational diabetic pregnancies. Early Hum Dev.

[11] Tabatabaei N, Giguère Y, Forest J-C, Rodd CJ, Kremer R, Weiler HA (2014). Osteocalcin is higher across pregnancy in Caucasian women with gestational diabetes mellitus. Can J Diabetes.

